# Supplementary material for: Robust antibiotic sensitization of pathogenic Pseudomonas aeruginosa via negative hysteresis in the cell envelope
Source: Nat Commun. 2026 Mar 26;17:4487. doi: 10.1038/s41467-026-71178-5 (PMC13187417; doi:10.1038/s41467-026-71178-5)
Supplement: Supplementary file 2 — Description of Additional Supplementary File [file 41467_2026_71178_MOESM2_ESM.pdf]

Description of Additional Supplementary Files

File Name: Supplementary Data 1
Description: Determination of minimum inhibitory concentrations of strains used in Figure 3

File Name: Supplementary Data 2
Description: Detailed statistical analysis of the pre-treatment effect for data used in Figure 3

File Name: Supplementary Data 3
Description: Detailed statistical analysis of the hysteresis response observed in Figure 3

File Name: Supplementary Data 4
Description: Transcriptome analysis of pre-treated and untreated PA14 WT at t0

File Name: Supplementary Data 5
Description: List of significantly differentially expressed genes of pre-treated and untreated PA14 WT at t0 with absolute fold change >3 and the KEGG analysis of significantly expressed genes of CpxS T163P at t0, t30, and t60 vs. PA14 WT with an absolute fold change of >3

File Name: Supplementary Data 6
Description: Transcriptome analysis of baseline expression of CpxS T163P at t0, t30, and t60 vs. PA14 WT

File Name: Supplementary Data 7
Description: List of significantly differentially expressed genes of CpxS T163P at t0, t30, and t60vs. PA14 WT with an absolute fold change of >2

File Name: Supplementary Data 8
Description: Overview of all strains used in this study

File Name: Supplementary Data 9
Description: Overview of all plasmids used in this study

File Name: Supplementary Data 10
Description: Overview of all antibiotics used in this study

File Name: Supplementary Data 11
Description: Additional information such as morphology and MIC of the individual patient population isolates

File Name: Supplementary Data 12
Description: A list of all primers used for the construction of mutants by two-step allelic exchange

File Name: Supplementary Data 13
Description: A list of primers used to create the inducible CpxS overexpression mutants
