# Supplementary material for: Robust antibiotic sensitization of pathogenic Pseudomonas aeruginosa via negative hysteresis in the cell envelope
Source: Nat Commun. 2026 Mar 26;17:4487. doi: 10.1038/s41467-026-71178-5 (PMC13187417; doi:10.1038/s41467-026-71178-5)
Supplement: Supplementary file 16 — Reporting summary [file 41467_2026_71178_MOESM16_ESM.pdf]

nature portfolio

Corresponding author(s): Hinrich Schulenburg

Last updated by author(s): Feb 16, 2026

Reporting Summary

Nature Portfolio wishes to improve the reproducibility of the work that we publish. This form provides structure for consistency and transparency in reporting. For further information on Nature Portfolio policies, see our Editorial Policies and the Editorial Policy Checklist.

Statistics

For all statistical analyses, confirm that the following items are present in the figure legend, table legend, main text, or Methods section.

n/a	Confirmed	
☐	☒	The exact sample size (*n*) for each experimental group/condition, given as a discrete number and unit of measurement
☐	☒	A statement on whether measurements were taken from distinct samples or whether the same sample was measured repeatedly
☐	☒	The statistical test(s) used AND whether they are one- or two-sided<br>*Only common tests should be described solely by name; describe more complex techniques in the Methods section.*
☒	☐	A description of all covariates tested
☐	☒	A description of any assumptions or corrections, such as tests of normality and adjustment for multiple comparisons
☐	☒	A full description of the statistical parameters including central tendency (e.g. means) or other basic estimates (e.g. regression coefficient) AND variation (e.g. standard deviation) or associated estimates of uncertainty (e.g. confidence intervals)
☐	☒	For null hypothesis testing, the test statistic (e.g. *F*, *t*, *r*) with confidence intervals, effect sizes, degrees of freedom and *P* value noted<br>*Give P values as exact values whenever suitable.*
☒	☐	For Bayesian analysis, information on the choice of priors and Markov chain Monte Carlo settings
☒	☐	For hierarchical and complex designs, identification of the appropriate level for tests and full reporting of outcomes
☐	☒	Estimates of effect sizes (e.g. Cohen's *d*, Pearson's *r*), indicating how they were calculated

Our web collection on statistics for biologists contains articles on many of the points above.

Software and code

Policy information about availability of computer code

Data collection	Software on plate-readers: Epoch 2, Agilent (GEN 5 v 3.08); Tecan infinite 200Pro (Tecan i-control v 2.0.10.0)
Data analysis	The data was analyzed using the platform R, version 3.6.1 - 4.2.2. Transcriptomics data was analysed with the following software packages: Fast QC (v0.11.9), MultiQC as part of Miniconda 3 (v4.8.2), Trimmomatic (v.0.39), Bowetie2 (v2.4.1), EDGE-pro (v1.3.1), edgeR (v3.28.1), cluterprofiler (v3.14.3)

For manuscripts utilizing custom algorithms or software that are central to the research but not yet described in published literature, software must be made available to editors and reviewers. We strongly encourage code deposition in a community repository (e.g. GitHub). See the Nature Portfolio guidelines for submitting code & software for further information.

Data

Policy information about availability of data

All manuscripts must include a data availability statement. This statement should provide the following information, where applicable:

- Accession codes, unique identifiers, or web links for publicly available datasets
- A description of any restrictions on data availability
- For clinical datasets or third party data, please ensure that the statement adheres to our policy

Phenotypic, and statistical data generated in this sudy are provided in the Supplementary Information - Datasets / Tables. Transcriptomic data generated in this study have been deposited in the NCBI's Gene Expression Omnibus under GEO Series acession number GSE290299

Research involving human participants, their data, or biological material

Policy information about studies with human participants or human data. See also policy information about sex, gender (identity/presentation), and sexual orientation and race, ethnicity and racism.

Reporting on sex and gender	Only microbial isolates from patients enrolled in this study were used. No other patient related data is provided in the manuscript.
Reporting on race, ethnicity, or other socially relevant groupings	Only microbial isolates from patients enrolled in this study were used. No other patient related data is provided in the manuscript.
Population characteristics	Adults with chronic obstructive pulmonary disease (COPD)/non-CF bronchiectasis. Sex: All, Minimum Age: 18, No Maximum Age. Inclusion Criteria: Dianosis of COPD and /or non-CF bronchiectasis with exacerbation; Detection of airway colonization by P. aerugionsa
Recruitment	Recruitment is ongoing. Recruitment countries: Germany, Number of Study centers: Multicenter study, Recruitment location(s): Medical center Forschungszentrum Borstel, Medizinische Klinik Borstel, University medical center UKSH (Campus Kiel), Medical center LungenClinic Grolssshansdorf, University medical center UKSH (Campus Lubeck). Adults with chronic obstructive pulmonary disease (COPD)/ non-CF bronchiectasis. Sex: All, Minimum Age: 18, No Maximum Age. Inclusion Criteria: Diagnosis of COPD and /or non-CF bronchiectasis with exacerbation; Detection of airway colonization by P. aeruginosa. We used 6 patient populations from four patients, two patients had two sample time points included, that were collected between January and November 2021.
Ethics oversight	Local ethics committee of the Medical Faculty of the University of Lubeck (No. 20-295),, available through the German Clinical Trials Register (https://drks.de/, ID DRKS00023975). Prior to enrollment, patients provided written informed consent for participation.

Note that full information on the approval of the study protocol must also be provided in the manuscript.

Field-specific reporting

Please select the one below that is the best fit for your research. If you are not sure, read the appropriate sections before making your selection.

☒ Life sciences ☐ Behavioural & social sciences ☐ Ecological, evolutionary & environmental sciences

For a reference copy of the document with all sections, see nature.com/documents/nr-reporting-summary-flat.pdf

Life sciences study design

All studies must disclose on these points even when the disclosure is negative.

Sample size	We did not perform a specific power analysis. For each experiment, we aimed at a sample size, which was as high as possible while still l allowing us to run all treatments and replicates in parallel and in randomized order.
Data exclusions	Pre-determined criteria were used for OD value quality control for the hysteresis screen, to avoid artifacts impacting the data points. Data was controlled for spikes in OD600 between the neighboring time points. We defined a cut-off of 0.05 change in OD600 to detect most spikes while minimizing false positives. Outlier OD600 values were identified by comparing each value against identical treatment replicates for a given time point. Outlier were flagged using a z-value > 3 and a distance of more than 3 interquartile ranges from the first and third quartile. Data points were omitted if both condition were true. Additionally, we removed all samples that did not include the first and last time points, as equal length is essential when calculating the area under the curve. All of this is explained in detail in the extended methods (see supplement 4.6)
Replication	Reproducibility of the findings was ensured by the use of independent biological replicates,except for the screen where we used technical replicates to determine hysteresis presence and biological replicates to determine robustness. The "Methods" section provides information on how often an experiment was performed. We included all relevant data in our analyses to address and statistically evaluate a particular question or hypothesis.
Randomization	For our experiments, the relevant treatment groups were run in parallel and in randomized set-ups.
Blinding	Blinding during data collection was achieved by masking expectations of each treatment group from the experimenter.

Reporting for specific materials, systems and methods

We require information from authors about some types of materials, experimental systems and methods used in many studies. Here, indicate whether each material, system or method listed is relevant to your study. If you are not sure if a list item applies to your research, read the appropriate section before selecting a response.

Materials & experimental systems

n/a	Involved in the study
☒	☐ Antibodies
☒	☐ Eukaryotic cell lines
☒	☐ Palaeontology and archaeology
☒	☐ Animals and other organisms
☒	☐ Clinical data
☒	☐ Dual use research of concern
☒	☐ Plants

Methods

n/a	Involved in the study
☒	☐ ChIP-seq
☒	☐ Flow cytometry
☒	☐ MRI-based neuroimaging

Plants

Seed stocks	no plants were used in this study
Novel plant genotypes	no plants were used in this study
Authentication	no plants were used in this study
